# Converging infectious disease threats in the post-COVID era: surveillance fragility, pandemic risk, and global preparedness

**DOI:** 10.3389/fpubh.2026.1916865

**Published:** 2026-09-04

**Authors:** Yusuf Hared Abdi, Zakaria Ibrahim Issack

**Affiliations:** 1 Department of Public Health, Faculty of Medicine and Health Sciences, Hormuud University, Mogadishu, Somalia; 2 Department of Health Emergency, One Health Unit, Ministry of Health and Human Services, Mogadishu, Somalia

**Keywords:** antimicrobial resistance, climate-sensitive diseases, infectious disease surveillance, one health, pandemic preparedness

## Abstract

The COVID-19 pandemic exposed profound weaknesses in global infectious disease surveillance yet, in its aftermath, pandemic-prone threats have intensified rather than receded. This narrative review synthesizes contemporary evidence on three converging drivers of pandemic risk zoonotic emergence, antimicrobial resistance, and climate-sensitive disease expansion and examines how structural failures in surveillance and global health governance amplify their impact. Drawing on recent epidemiological analyses, policy reports, and One Health evaluations, the review maps post-COVID surges in zoonotic outbreaks, drug-resistant infections, and vector and water-borne diseases, and links these trends to fragmented laboratory networks, inequitable genomic capacity, and weak integration across human, animal, and environmental systems. It further interrogates the geopolitical and socioeconomic determinants of vulnerability, including conflict-related surveillance collapse, vaccine inequity, and the political economy of pandemic financing. Emerging digital tools from AI-enabled forecasting to wastewater and social media surveillance are assessed not only for their technical promise but also for risks related to data bias, extractives, and governance gaps. The review argues that without redistributive investment in One Health integration, regional genomic platforms, community-based surveillance, and enforceable IHR-based accountability, the world will continue to detect converging threats late and respond inequitably, leaving pandemic preparedness structurally fragile in the post-COVID era.

## 1 Introduction

The COVID-19 pandemic stands as the most consequential infectious disease event of the 21st century. As of March 2024, it had produced approximately 774.8 million confirmed cases and 7 million official deaths globally (1). Three years after peak COVID-19 mortality, the Global Preparedness Monitoring Board of the WHO and World Bank described global pandemic preparedness as remaining “fragile.” Political leaders, distracted by geopolitical frictions and lulled by the easing of COVID-19 death rates, are neglecting the pandemic’s grim lessons, even as new threats loom (2). In 2024, the number of pandemic-prone and epidemic-prone disease outbreaks worldwide was estimated at 301, with approximately 90% associated with COVID-19, dengue, yellow fever, Oropouche virus disease, and influenza (3). A June 2024 analysis by Airfinity and Bloomberg News, drawing on data from over 60 organizations and public health agencies, found that at least 13 infectious diseases are showing a resurgence post-pandemic, with more than 40 countries or territories reporting at least one infectious disease resurgence at 10-fold or more over their pre-pandemic baseline (4).

The key drivers of this accelerating burden are well documented. First, falling vaccination rates left populations exposed to vaccine-preventable diseases, with 20 countries in Europe dropping below 90% measles vaccination coverage in 2022 (4). Second, the COVID-19 pandemic caused disruptions to routine immunization programs in at least 68 countries, affecting more than 80 million children worldwide (5). Third, climate change, deforestation, urbanization, and agricultural intensification are expanding the ecosphere in which pathogens can emerge and spread (6). The convergence of these forces ecological disruption, healthcare system strain, post-pandemic fatigue, and structural inequality creates the precise conditions for the next major outbreak (5). A 2024 survey of 103 epidemiologists and virologists by the Abbott Pandemic Defense Coalition found that 75% believe the next major global health challenge will likely occur in the next 5 to 25 years, and that disease surveillance remains the most important tool for identifying the next pandemic. Viral surveillance and wastewater surveillance were identified as the top two investment priorities. These experts also flagged misinformation, distrust in public health officials, and a lack of funding for public health institutions as the primary barriers to sustained readiness (7). Post-COVID reforms including the WHO Hub for Pandemic and Epidemic Intelligence in Berlin, the Pandemic Fund established jointly with the World Bank, and the Global Health Emergency Corps represent genuine institutional progress. Yet structural inequalities in laboratory infrastructure, genomic surveillance capacity, and One Health integration ensure that the next major pathogen will again be detected late, understood slowly, and responded to inequitably (8). The sections that follow examine the specific threats, the surveillance failures that leave them inadequately detected, and the analytical dimensions of this ongoing global vulnerability.

Despite the scale of these threats, few integrative narrative reviews have examined zoonotic emergence, antimicrobial resistance, and climate-driven disease expansion alongside the specific surveillance failures and geopolitical vulnerabilities that collectively shape pandemic risk in the post-COVID era. Existing literature tends to address these domains in isolation, leaving the critical interconnections particularly the compounding mechanisms of threat convergence and the equity dimensions of governance reform underexplored. Therefore, this narrative review aims to explore the intersections of these converging infectious disease threats, analyze the structural failures in global surveillance systems, and highlight strategic priorities for strengthening pandemic preparedness with equity at the center.

## 2 Methodology

To construct this integrative narrative review, a targeted literature search was conducted to identify peer-reviewed articles, epidemiological reports, and global health policy documents. The primary search timeframe focused on literature published between January 2020 and June 2026 to capture post-COVID-19 trends; however, foundational studies and critical historical analyses (e.g., pre-pandemic Ebola and Hantavirus outbreaks) published outside this period (2010–2019) were intentionally included to provide necessary epidemiological and ecological context. Databases consulted included PubMed, Scopus, and Web of Science, supplemented by grey literature from the institutional repositories of the World Health Organization (WHO), Africa CDC, and the World Bank.

The search strategy utilized combinations of the following keywords: (“pandemic preparedness” OR “global health security”) AND (“infectious disease surveillance” OR “One Health” OR “genomic surveillance”) AND (“zoonotic diseases” OR “antimicrobial resistance” OR “climate-sensitive diseases”). Initial identification involved screening article titles and abstracts for relevance, restricted to English-language publications. Articles were then selected for full-text review if they provided empirical data, epidemiological trends, or policy analyses related to post-COVID-19 surveillance infrastructure, digital epidemiology, or the geopolitical determinants of outbreak response. Additionally, a manual backward citation search (snowballing) of the reference lists of included articles was performed to identify further relevant sources that did not appear in the initial database queries.

Studies focusing exclusively on the clinical management of specific pathogens without a public health or surveillance context were excluded. The synthesized literature was then thematically organized to analyze three intersecting domains: the biological drivers of outbreak risk, the structural fragmentation of current surveillance systems, and the geopolitical dynamics influencing global health governance.

## 3 The evolving landscape of global infectious threats

### 3.1 Emerging and re-emerging zoonotic diseases

Zoonotic diseases those transmitted from animals to humans are responsible for an estimated 2.5 billion illnesses and 2.7 million deaths annually, representing nearly 60% of all infectious diseases and 75% of all newly emerging infections (9). The COVID-19 pandemic has set the benchmark for potential future damage, but the biological preconditions for an equally devastating or worse event remain firmly in place (9).

H5N1 avian influenza has become one of the most geographically expansive and biologically dynamic threats in the current global health landscape. Since late 2020, clade 2.3.4.4b strains have dominated outbreaks across multiple continents (10). Critically, human cases have continued to occur. In the U.S., an outbreak in dairy cattle first detected in March 2024 resulted in human cases (11). Between September and November 2025, 19 human HPAI infections were reported across four countries, including two deaths (12).

The re-emergence of monkeypox (mpox) has been one of the defining infectious disease stories of the 2020s. Following the 2022 global outbreak declared a PHEIC in July 2022 a new and more virulent strain emerged. In August 2024, WHO Director-General Dr. Tedros Adhanom Ghebreyesus declared the upsurge of mpox in the Democratic Republic of the Congo (DRC) and neighboring African countries a second PHEIC, based on the rapid spread of a new clade 1b strain (13). As of September 2024, more than 29,000 cases had been reported with over 800 fatalities, a case fatality rate of approximately 3%. The WHO emphasized the risk of global spread, calling the situation “an emergency not only for Africa, but for the entire globe” (13). Beyond currently circulating threats, novel coronaviruses and other uncharacterized pathogens remain a standing concern. COVID-19 caused a 25% increase in the global prevalence of anxiety and depression in the first year of the pandemic and disrupted education for an entire generation of children in parts of the Global South (5). The WHO has identified over 30 priority pathogens that could cause future global public health emergencies (6).

The conditions that produce zoonotic emergence are deepening, not resolving. The wildland-urban interface (WUI) covering just 5% of Earth’s land surface is a prime zone for disease spillover, with nearly 3.5 billion people living at the boundary between cities and wild places (14). A 2025 Yale School of the Environment study mapping wildlife host distribution across the global WUI found that over 700 million people live in areas with suitable habitat for 20 or more zoonotic host species (15). The interplay of human behavior and environmental encroachment is further exemplified by the emergence of Hantavirus and other pathogens linked to expanding ecotourism and travel into previously isolated habitats^1^. Similarly, recent outbreaks of Ebola virus disease in the DRC underscore how emerging viral threats are fatally compounded by armed conflict and profound community distrust in health systems, allowing localized spillover events to rapidly escalate (doi: 10.1073/pnas.1913980116). In the next 25 years, urban populations will increase by 2.5 billion, with 90% of that growth predicted in Africa and Asia precisely the regions with the highest biodiversity, poorest infrastructure, and greatest undetected pathogen diversity (14). Deforestation, agricultural intensification, informal urbanization, and wildlife trade are together expanding the ecological frontiers at which pathogens move into human populations (9).

### 3.2 Antimicrobial resistance: the silent pandemic

Antimicrobial resistance (AMR) is not a future threat, it is a present and escalating crisis. The WHO warns that without urgent action, AMR could cause 10 million deaths annually by 2050, surpassing cancer-related fatalities and threatening to rival the global burden of major non-communicable diseases. Already, AMR directly causes more deaths globally than major communicable diseases like HIV/AIDS and malaria combined (doi: 10.1016/S0140-6736(21)02724-0) (16). In the interim, drug-resistant infections are already complicating routine surgeries, cancer chemotherapy, and organ transplantation (17). Among bacterial pathogens, tuberculosis remains the world’s leading cause of death from an infectious disease, claiming 1.25 million lives in 2023, despite a cure existing for over 70 years (18). The challenge is intensified by the spread of drug resistance. In 2023, an estimated 400,000 people developed MDR-TB or rifampicin-resistant TB globally, and approximately 150,000 died (18). MDR-TB has been reported in virtually every country in the world (16). Extensively drug-resistant TB (XDR-TB) resistant to four commonly used anti-TB drugs has been confirmed in over 127 countries and represents approximately 5% of all MDR-TB cases (19). Only 2 in 5 people in need of DR-TB treatment were diagnosed and started on treatment in 2023 (16). In 2024, only 14% of the estimated 30,000 children with MDR/RR-TB were diagnosed and initiated on treatment (18). The treatment burden is severe: traditional regimens require up to 14,000 pills per patient and can last 2 years (19).

Hospital-acquired resistance presents an equally urgent challenge. Carbapenem-resistant Enterobacterales (CRE) are designated by WHO as critical-priority pathogens (20). A multi-site study across Ethiopia, Greece, and India found that 22% of 14,078 Enterobacterales isolates were carbapenem-resistant, with *Klebsiella pneumoniae* resistance rates of 42–54% (21). Alarmingly, resistance is increasingly being reported against WHO “Reserve” group antibiotics such as colistin and cefiderocol which are intended as last-resort options for life-threatening multi-drug resistant infections in healthcare settings (doi: 10.1016/S0140-6736(21)02724-0). In one Saudi Arabian tertiary hospital study of CRE infections, 90-day mortality reached 42.6% (20). Hospital-acquired infections affect between 5 and 15% of hospitalized patients worldwide, with higher prevalence in ICUs and resource-limited settings (22). The ESKAPE pathogens (*Enterococcus faecium*, *Staphylococcus aureus*, *Klebsiella pneumoniae*, *Acinetobacter baumannii*, *Pseudomonas aeruginosa*, and *Enterobacter* species) are responsible for the majority of life-threatening hospital-acquired infections and are notable for their capacity to escape the effects of antimicrobial agents (23).

Drug resistance is also eroding the treatment of parasitic disease. The cornerstone of malaria treatment globally is artemisinin-based combination therapy (ACT). Artemisinin partial resistance, first recognized in Southeast Asia, has now emerged *de novo* in East Africa in Rwanda, Uganda, South Sudan, Tanzania, Ethiopia, Eritrea, and eastern DRC (24). As of 2024, this represents an ominous shift: ACTs are the most widely used antimalarial drugs in the world, and their failure would dramatically increase malaria deaths (25). In 2024, there were an estimated 282 million malaria cases and 610,000 deaths globally, with 95% of deaths in the WHO African Region, mostly among children under 5 (26). New prevention tools including dual-ingredient bed nets and WHO-recommended vaccines prevented an estimated 170 million cases and 1 million deaths in 2024, but antimalarial drug resistance now threatens to erode this progress (26).

The total number of antibacterial agents in clinical development grew from 80 in 2021 to 97 in 2023, but only 12 of the 32 antibiotics targeting WHO’s Bacterial Priority Pathogen List (BPPL) infections can be considered innovative (27). Only 4 of these 12 are active against at least one WHO ‘critical’ priority pathogen. Since July 2017, 13 new antibiotics obtained marketing authorization globally, but only 2 represent a new chemical class (27). The pipeline is described by WHO officials as insufficient: “Antimicrobial resistance is only getting worse yet we’re not developing new trailblazing products fast enough”. Weak commercial incentives, high regulatory barriers, and the global overuse and misuse of antibiotics in human medicine and livestock production perpetuate this crisis (28). Antibiotic stewardship programs remain weak and fragmented across low and middle-income countries (LMICs), where diagnostic capacity for identifying resistant organisms is limited and regulatory oversight of antibiotic sales is often inadequate (29). In high-burden settings, AMR surveillance is further hampered by the absence of standardized reporting systems, limited laboratory infrastructure, and insufficient trained personnel (18).

### 3.3 Climate change and infectious disease expansion

The WHO World Malaria Report 2023 highlighted that climate change is the single biggest health threat facing humanity, citing the 2022 Pakistan floods that produced a fivefold increase in malaria cases 2.1 million more than the previous year (30). More than 60% of infectious disease epidemiologists surveyed by the Abbott Pandemic Defense Coalition identified mosquito-borne diseases as the biggest threat to public health as the climate changes, citing dengue, Zika, and West Nile virus as priority concerns (7). In 2024, 14.1 million dengue cases were reported globally a twofold increase from 2023 and a 12-fold rise compared to 2014 (31). In the United States, dengue cases jumped 359% in 2024 compared to the annual average from 2010 to 2023 (32). The 2024 surge reflects both the growing climate sensitivity of dengue virus transmission and the expanding presence of *Aedes* mosquitoes in urban environments at higher latitudes and altitudes (33). By 2025, 3.6 million dengue cases were already reported across 94 countries in the first part of the year (34).

Climate change is producing conditions that support the geographic expansion of *Anopheles* and *Aedes* mosquito populations. Malaria transmission is expected to shift poleward and to higher altitudes, while the dengue and chikungunya vector (*Aedes aegypti* and *Aedes albopictus*) continues to establish itself in previously non-endemic temperate zones. The WHO and its partners have identified that these shifts will disproportionately affect communities already burdened by poverty, inadequate health systems, and climate vulnerability including those across sub-Saharan Africa and South Asia (35).

Cholera presents a parallel trajectory. In 2024, the WHO reported more than 560,000 cholera cases and over 6,000 deaths from 60 countries, a 5% increase in cases and a 50% rise in deaths compared to 2023 (36). The number of countries affected has almost doubled since 2017, and the number of very large outbreaks has tripled (37). Conflict, climate change, population displacement, and chronic deficiencies in water, sanitation, and hygiene (WASH) infrastructure continue to fuel the surge (36). Africa and the Caribbean account for 98% of all reported cholera cases globally, reflecting the convergence of climate exposure and infrastructural fragility (36).

### 3.4 The mechanisms of convergence: a conceptual framework

While zoonoses, antimicrobial resistance, and climate-sensitive diseases are often tracked through separate epidemiological silos, their true threat lies in their convergence. These three drivers do not operate in isolation; rather, they interact through shared upstream determinants and compounding biological mechanisms, creating a “syndemic” risk profile that traditional, single-disease surveillance systems are ill-equipped to detect.

Conceptually, this convergence operates through intersecting pathways of ecological disruption, pathogen evolution, and infrastructural strain. For example, agricultural intensification a primary driver of deforestation simultaneously increases human-wildlife contact (facilitating zoonotic spillover) and relies heavily on the prophylactic use of antibiotics in livestock, which accelerates the environmental spread of AMR^2^. Similarly, climate change directly amplifies AMR risk: extreme weather events and severe flooding displace populations and destroy fragile Water, Sanitation, and Hygiene (WASH) infrastructure, creating the ideal vectors for the rapid dissemination of highly resistant, waterborne bacterial pathogens like cholera and typhoid^3^.

Furthermore, these converging biological threats systematically target the same geopolitical vulnerabilities. A climate-driven surge in dengue fever can rapidly overwhelm a fragile health system’s diagnostic capacity, leaving that same population blinded to a concurrent zoonotic spillover or an outbreak of a drug-resistant hospital-acquired infection. By viewing global health security through this convergence framework, it becomes evident that a reactive, pathogen-specific approach to surveillance is structurally inadequate. Anticipating the next pandemic requires an analytical lens that monitors the intersections of ecological pressure, microbial resistance, and human vulnerability simultaneously.

## 4 Structural challenges in country and region-based surveillance systems

Before examining systemic vulnerabilities, it is crucial to recognize the significant successes and good practices in disease surveillance across both high-income countries (HICs) and low and middle-income countries (LMICs). For example, the United Kingdom’s genomic sequencing consortium (COG-UK) (doi: 10.1016/S2666-5247(20)30054-9) and the European Union’s widespread wastewater monitoring networks (doi: 10.3390/v16091398) have demonstrated how highly integrated, well-funded systems can track pathogen evolution and community spread in near real-time. In resource-limited settings, the successful repurposing of the Global Polio Eradication Initiative’s surveillance infrastructure in countries like Nigeria and Pakistan to track COVID-19 and other infectious diseases showcases how existing community, logistical, and laboratory networks can be efficiently leveraged (doi: 10.4314/mmj.v34i4.12). These working models provide critical blueprints for global health security. However, significant challenges remain in scaling these successes equitably across regions.

### 4.1 Fragmentation of international surveillance networks

Sub-Saharan Africa, which accounts for nearly 32% of all global disease outbreaks since 1996, has historically been severely underserved in laboratory diagnostic capacity (3). Before the COVID-19 pandemic, only seven African countries had the capacity to sequence pathogens locally; samples had to be sent overseas, resulting in delays that rendered sequences far less actionable for public health (38). The African Pathogen Genomics Initiative, accelerated by COVID-19, increased this to 35 countries with sequencing capacity in just the first year, and by 2025, 39 African countries have machinery, computing power, reagents, and trained personnel to perform in-country sequencing (38). Despite this progress, Sub-Saharan Africa has made strides, with more than 50% of countries now equipped with in-country sequencing capacity (39), but Central Africa still has significant surveillance facility gaps (40). Unequal access to testing reagents, bioinformatics support, and stable electricity supply limits the practical utility of available hardware in many countries. Traditional surveillance systems are increasingly limited by delayed reporting, data silos, and fragmented architectures that prevent real-time situational awareness (41). Partners exploring wastewater surveillance are now tracking not just polio, but hepatitis E, measles, and mpox, with promising results. The South African National Institute for Communicable Diseases recently detected a full measles genome in wastewater in an area with no reported clinical cases, enabling early outbreak mapping. Scaling this capacity equitably particularly in informally-sewered communities remains a major technical and infrastructural challenge.

### 4.2 Weak integration between human, animal, and environmental surveillance

The WHO defines One Health as an approach that, over time, can improve prevention, reduce fragmentation, and support more efficient use of limited resources (42). In practice, however, its implementation is severely limited by institutional fragmentation: ministries of health, agriculture, environment, and veterinary services operate in separate bureaucratic silos, with different funding streams, reporting mechanisms, and professional cultures (43). In India’s ambitious “One Nation, One Health System” policy targeting 2030 the focus has been on intra-human health systems integration rather than the inter-sector integration of human, animal, and ecosystem health demanded by One Health principles (44). Across most nations, veterinary services and public health departments maintain parallel, non-interoperable data systems with minimal joint planning for zoonotic disease surveillance (44). A systematic review of 202 One Health systems published between 2015 and 2024 found that only 30% integrate human and animal data, 12% combine human and environmental data, and fully integrated systems incorporating all three domains remain rare (45). Most of these integrated systems are commentary pieces rather than functional, operational tools (45). Wildlife trade data from CITES, agricultural disease databases, and human health surveillance registries are rarely linked, even though the wildlife trade is among the most potent early indicators of zoonotic spillover risk (46). This absence of interoperable databases means that the signature of an emerging zoonotic threat patterns of unusual animal die-offs, veterinary anomalies, or environmental contamination are typically invisible to human public health surveillance until disease has already crossed into human populations (47).

High Pathogenicity Avian Influenza (HPAI) in sub-Saharan Africa is actively monitored by FAO across countries including Nigeria, South Africa, Senegal, and DRC, with novel reassortant genotypes being identified that did not originate from Europe suggesting endogenous evolutionary dynamics previously overlooked (48). The response capacity of national veterinary authorities varies enormously, and coordination with human health surveillance systems for the same pathogens is inconsistent and often informal (49).

The 2025 ECOWAS-supported One Health FELTP Frontline program in The Gambia, which recruited participants from human health, veterinary, and environmental sectors for joint zoonotic outbreak investigation training, represents the type of cross-sectoral capacity building that One Health demands (50). The FAO/UNEP/WHO/WOAH One Health Joint Plan of Action explicitly acknowledges that “multidisciplinary barriers, a lack of resources, and a lack of transparency for information-sharing have impeded the implementation of One Health in practice” (51).

Zoonotic diseases are emerging at the wildlife-human interface with increasing frequency, driven by ecological disruption, urbanization, and agricultural intensification (14). AMR is eroding the efficacy of the antibiotics, antimalarials, and antituberculosis agents that form the backbone of infectious disease treatment in every health system (16). Climate change is expanding the geographic range of vector-borne diseases, producing conditions favorable to cholera and other waterborne diseases, and increasing the unpredictability of outbreak timing and severity (36).

Against this backdrop, global surveillance systems remain fragmented, underfunded, inequitably distributed, and politically vulnerable to complacency (41). Sub-Saharan Africa which bears a disproportionate burden of emerging infectious disease still lacks the laboratory infrastructure, genomic sequencing capacity, and interoperable One Health data systems needed to detect, characterize, and report outbreaks with the speed required to prevent global spread (39). The IHR reporting framework, wastewater surveillance networks, and the One Health Joint Plan of Action represent credible building blocks for a stronger architecture but only if backed by sustained political commitment and equitable investment in LMIC health security capacity (8). The lessons of COVID-19, Ebola, mpox, H5N1, and cholera are not merely historical. They are active, ongoing, and worsening. The question is not whether the world will face another major outbreak, it is whether, when that moment arrives, the infrastructure will exist to detect it quickly enough, characterize it accurately enough, and respond equitably enough to prevent it from becoming the next pandemic.

## 5 Digital transformation of infectious disease surveillance

### 5.1 Artificial intelligence and predictive epidemiology

Artificial intelligence is rapidly reshaping the landscape of infectious disease forecasting. A landmark 2025 Nature perspective paper led by researchers from the University of Oxford and partners across five continents outlined, for the first time, how AI can transform pandemic preparedness from modelling disease spread and pinpointing high-transmission zones to predicting which new variants might arise and whether cross-species jumps are likely (52). In 2025, a Johns Hopkins and Duke University team published PandemicLLM in *Nature Computational Science* the first tool to use large language modelling for infectious disease forecasting, integrating epidemiological time-series, genomic surveillance, public health policy data, and spatial demographics to predict hospitalization trends one to 3 weeks ahead, consistently outperforming models on the CDC’s CovidHub (53). Emerging AI models show promise in forecasting disease spread and identifying outbreak patterns earlier than traditional surveillance. However, critical ethical concerns temper this optimism. AI performance depends on the quality and representativeness of training data—a particular concern since models trained predominantly on high-income country data may produce unreliable forecasts for low-income countries where data are sparse or fragmented (54).

However, critical ethical and structural concerns temper this optimism. The Oxford study authors explicitly warned that AI performance depends on the quality and representativeness of training data, noting that the limited accessibility of AI models to the wider scientific community, and the risks of deploying “black-box” models for public health decision-making, pose serious problems (55). Algorithmic bias is a particular concern: models trained predominantly on data from high-income settings may produce unreliable forecasts for low-income countries where data are sparse, fragmented, or systematically under collected (56). Questions of data ownership who controls the vast streams of genomic, clinical, and mobility data that feed AI systems intersect with the broader concern about data extractivism from disease-burdened LMICs (55).

### 5.2 Digital epidemiology and social media surveillance

Digital surveillance leveraging data from social media platforms, search engines, and online health portals has emerged as a powerful early warning tool for infectious disease outbreaks. Analyses of Twitter, Google Trends, and other platforms have demonstrated the ability to detect outbreak signals days to weeks before formal reporting systems trigger alerts (57). Social media data provide real-time windows into symptom prevalence, health-seeking behavior, and community anxiety making them valuable complements to conventional sentinel surveillance (58).

Yet the same digital ecosystem that enables early outbreak detection also amplifies misinformation at unprecedented speed. The WHO defines an “infodemic” as too much information including false or misleading content during a disease outbreak, which causes confusion, risk-taking behaviors, and erosion of trust in health authorities (59). During COVID-19, infodemics competed directly with public health messaging, undermining vaccination uptake and distorting risk perception on a global scale (59). The WHO has since developed infodemic management tools including EARS (an early AI-supported social listening platform), a repository of 200 fact-checking groups across 40 languages, and an AI-based infodemic observatory to counteract the structural vulnerability that digital surveillance also creates. Managing the dual-use nature of digital epidemiology harnessing outbreak signals while countering misinformation amplification remains one of the most pressing governance challenges in contemporary global health security.

## 6 Geopolitical and socioeconomic drivers of outbreak vulnerability

### 6.1 Conflict, displacement, and fragile health systems

Armed conflict is one of the most reliable predictors of catastrophic surveillance collapse. When health systems are targeted, displaced, or defunded, the epidemiological infrastructure required to detect, confirm, and respond to outbreaks disintegrate and humanitarian settings become epidemic accelerators. The recent Ebola outbreaks in the Democratic Republic of the Congo (DRC) vividly illustrate this dynamic; armed group violence targeting health facilities, coupled with deep-seated community distrust, severely hampered contact tracing, vaccination campaigns, and epidemiological surveillance, allowing the virus to spread unchecked (doi: 10.1073/pnas.1913980116). Sudan similarly exemplifies the convergence: 2 years into the conflict, more than two-thirds of states are battling multiple outbreaks cholera, dengue, measles, malaria fuelled by surveillance collapse, disrupted vaccinations, and destroyed water systems. In April 2025, 156 healthcare attacks had killed 318 people, directly eroding remaining capacity. Conflict-driven surveillance collapse creates precise conditions for epidemics to accelerate unchecked (60).

### 6.2 Vaccine inequity and Global Health power asymmetries

The COVID-19 pandemic laid bare the structural asymmetries in global vaccine access with devastating clarity. Within the first year of COVID-19 vaccine distribution, high-income countries achieved vaccination rates of 75–80%, while low-income countries vaccinated fewer than 10% of their populations (61). The COVAX facility designed to guarantee equitable access was undermined from the outset: by mid-2021, COVAX had shipped just 30 million doses to 54 countries, falling catastrophically short of its goal of 1.8 billion doses for 92 LMICs by end-2021, partly due to high-income countries placing bilateral orders that bypassed the facility and partly due to chronic underfunding (62).

The TRIPS waiver debate initiated by South Africa and India at the WTO in 2020 to allow generic manufacturers in developing countries to produce COVID-19 vaccines without IP constraints highlighted the depth of the political resistance. These power asymmetries are not incidental features of the global health architecture, they are structural, recurring, and deeply consequential for future pandemic response equity.

Table 1 summarizes cross-cutting structural weaknesses in global infectious disease surveillance and outlines priority strategic reforms.

**Table 1:** Key weaknesses in current global infectious disease surveillance systems and recommended strategic reforms.

Current weakness	Consequence	Strategic reform
Delayed outbreak reporting	Rapid international spread before containment measures activate	Real-time interoperable reporting under strengthened IHR frameworks
Weak and inequitable genomic surveillance globally, particularly in LMICs	Delayed variant detection; missed pathogen evolution	Expansion of regional sequencing hubs through Africa CDC, PAHO, ASEAN, and equivalent platforms globally
Fragmented One Health systems	Missed zoonotic threats at animal-human-environment interfaces	Integrated multi-sector surveillance platforms with shared databases
Vaccine inequity and IP barriers	Uneven outbreak control; prolonged pandemic duration	Regional manufacturing expansion and TRIPS waiver implementation
Underfunded public health systems	Poor outbreak containment, especially in fragile states	Sustainable preparedness financing through the pandemic fund and national commitments

## 7 Strategic priorities for global response

### 7.1 Building integrated one health surveillance systems

The most durable solution to the fragmentation that characterizes current global surveillance is the genuine operationalization of One Health architecture. Integrated human-animal-environmental surveillance platforms must share data across sectors in real time, replacing the current siloed parallel systems in which veterinary, environmental, and human health databases rarely communicate (42). The WHO’s One Health Joint Plan of Action (2022–2026) provides a policy framework, but its four thematic tracks zoonoses, food safety, AMR, and environment require cross-sectoral governance models that move beyond voluntary coordination to institutionalized structures with dedicated funding streams (42). A 2025 systematic review spanning both high-income countries and LMICs found that only 30% of 202 One Health systems reviewed integrate human and animal data, illustrating how far implementation lags behind the conceptual framework (45). Priority investments include shared species-specific alert thresholds that trigger concurrent human and veterinary investigation; interoperable national databases accessible to both public health and agricultural ministries; and joint training programs like the ECOWAS One Health FELTP model in The Gambia that build cross-sectoral professional culture at the frontline (50).

### 7.2 Expanding regional genomic surveillance capacity

Africa CDC has led one of the most remarkable capacity expansions in the history of public health infrastructure. Between 2020 and 2026, the number of African Union member states with genomic sequencing capacity grew from 7 to 46 a transformation driven by the Africa Pathogen Genomics Initiative (Africa PGI) and supported by partnerships with the African Society for Laboratory Medicine (ASLM), the Mastercard Foundation, and the European Commission‘s HERA (63). More than 1,600 scientists have been trained through over 90 in-person genomics and bioinformatics programs between 2020 and 2025, and over 6,000 sequences have been shared through platforms including Terra (63). In 2024, Africa CDC launched two EU-co-funded flagship programs the Integrated Genomic Surveillance and Data Sharing Platform (IGS) and the Integrated Genomic Surveillance for Outbreak Detection (DETECT) to extend AMR genomic surveillance and accelerate data sharing across AU member states (64). For these gains to be durable, open-access genomic data platforms must become the norm, ensuring that sequences generated in Africa remain accessible to African scientists and inform African public health decisions rather than being absorbed into proprietary databases in high-income countries (39). This model of regional capacity building is equally vital and progressing in other parts of the world. In the Americas, the Pan American Health Organization (PAHO) has significantly expanded the COVID-19 Genomic Surveillance Regional Network (COVIGEN) to track broader emerging respiratory viruses and arboviruses like dengue across South and Central America^4^. Similarly, in Asia, initiatives like the Indian SARS-CoV-2 Genomics Consortium (INSACOG) have evolved to monitor broader infectious threats, (doi: 10.3389/fpubh.2023.1117602) while the Association of Southeast Asian Nations (ASEAN) is advancing regional data-sharing mechanisms for cross-border disease surveillance (doi: 10.1016/j.pdisas.2020.100080). Replicating and funding these regional networks is critical to decentering global surveillance architecture and ensuring rapid, locally-led responses.

### 7.3 Sustainable pandemic financing

The Pandemic Fund launched in November 2022 by G20 leaders and hosted by the World Bank with WHO as technical lead represents the most significant structural financial commitment to LMIC pandemic preparedness since COVID-19. Through three funding rounds, the Pandemic Fund has awarded grants totaling USD 1.4 billion, mobilizing an additional USD 10.1 billion in co-financing and co-investment across 128 countries in six geographic regions (65). Its third funding round, approved in December 2024, committed a $500 million grant envelope to support disease surveillance, diagnostics, laboratory systems, and health workforce capacity in LMICs (66). Despite this progress, demand for funding far outstrips available resources, with the Fund’s short-term fundraising goal of USD 2 billion still only halfway met as of early 2025 (66). Sustainable preparedness financing must go beyond episodic donor campaigns: it requires reliable multi-year commitments from both donor governments and recipient countries, integration of health security into domestic budget frameworks, and mechanisms to ensure financing continuity between rather than only during pandemic emergencies (65).

### 7.4 Strengthening community-based surveillance

Community-based surveillance (CBS) the systematic detection and reporting of health events of public significance within communities by community members themselves is the essential bridge between population-level disease events and formal health system notification chains (67). A 2025 Journal of Global Health study found that CBS for emerging infectious diseases provides a decentralized approach that allows communities to rapidly detect and report outbreak signals, particularly in settings where healthcare facilities are distant, under-resourced, or distrusted (68). Community health workers (CHWs) trained in event-based surveillance, contact tracing, and risk communication have been demonstrated to significantly improve early outbreak warnings with programs in West Africa, South Asia, and the Pacific documenting improved community trust and higher engagement in surveillance activities following CHW training (69). The critical challenge is integrating community-generated intelligence into national surveillance data flows so that anomalous symptom clusters, animal die-offs, or environmental events reported by communities translate in real time into alert signals that health authorities can act on, rather than being lost in informal channels (68).

### 7.5 Reforming international health governance

The 2024 amendments to the International Health Regulations adopted by consensus at the 77th World Health Assembly in Geneva and entering into force on 19 September 2025 represent the most significant reform to the legal architecture of global health governance since the IHR’s adoption in 2005 (70). The amendments introduce a new “pandemic emergency” alert level above PHEIC to trigger stronger international collaboration, mandate the establishment of National IHR Authorities to coordinate implementation, and include provisions on equitable access to medical products during emergencies (70). A BMJ Global Health analysis described the new National IHR Authorities as potentially transformative for weak intersectoral collaboration and insufficient authority to report public health events two of the most persistent barriers to IHR compliance (71).

However, critical structural limits persist. Under the IHR, WHO serves as Secretariat without authority to compel action by countries meaning that even with the 2024 amendments, reporting remains ultimately voluntary, and enforcement depends on political will (70). Eleven of the 196 IHR States Parties rejected the 2024 amendments outright (70), and the broader WHO Pandemic Agreement under negotiation in parallel still faces contentious debates over pathogen access, benefit sharing, and the balance between national sovereignty and global accountability. Political support and technical guidance will be critical to translate the legal framework into functional national systems particularly in LMICs where institutional capacity gaps remain the binding constraint on IHR compliance. Achieving enforceable accountability mechanisms whether through graduated incentive structures, conditionality within global health financing, or peer-review compliance systems remains the most unresolved challenge in international health governance reform.

## 8 Future directions

Can global surveillance evolve from reactive detection to predictive prevention?

The most consequential question in pandemic preparedness today is whether the world can fundamentally shift its surveillance paradigm from a system that detects outbreaks after they have begun to one that anticipates them before they cross critical thresholds. The convergence of several emerging technological domains makes this transition, for the first time, scientifically plausible.

AI-driven biosurveillance is moving from hypothesis to deployment. The Johns Hopkins–Duke PandemicLLM tool the first large language model applied to infectious disease forecasting demonstrated the ability to predict hospitalization trends one to 3 weeks ahead by integrating epidemiological, genomic, climate, and policy data streams simultaneously (53). A 2025 Nature Communications Perspective identified adoptability and sustainability not technical capacity as the primary barriers to implementing climate-informed prediction tools in national surveillance systems, underlining that technological innovation must be paired with governance and health system integration to realize its potential (72). Deep learning frameworks combining climate, epidemiological, and socioeconomic data are already demonstrating the ability to forecast endemic disease outbreaks up to 100 weeks in advance with reliable probabilistic intervals, offering a deployable early-warning layer particularly relevant to vector-borne and climate-sensitive diseases.

Precision public health the application of genomic, spatial, environmental, and behavioral data to individualize and target outbreak interventions represents a next-generation evolution beyond population-level surveillance. A 2024 scoping review in SAGE Journals described precision public health as capable of delivering more targeted and efficient responses by stratifying risk at granular geographic and demographic levels, enabling pre-emptive interventions that population-wide traditional surveillance cannot achieve (73).

## 9 Conclusion

The global infectious disease landscape is becoming more complex, more unpredictable, and more interconnected with each passing year. The failures exposed by COVID-19, Ebola, mpox, H5N1, and cholera have not yet produced a commensurate structural response. The next major outbreak will not announce itself in advance. Whether the global community can detect it early enough, characterize it accurately enough, and respond equitably enough to prevent catastrophe depends entirely on decisions taken in laboratories, legislatures, and multilateral forums before that moment arrives.

## Footnotes

1
https://pubmed.ncbi.nlm.nih.gov/23169317/

2
https://www.google.com/url?sa=E&q=https%3A%2F%2Fdoi.org%2F10.1038%2Fs41893-019-0293-3

3
https://www.unep.org/resources/superbugs/environmental-action

4
https://www.paho.org/en/documents/resolution-csp30r9-strategy-regional-genomic-surveillance-epidemic-and-pandemic
